# Cloud and Precipitation Profiling Radars: The First Combined W- and K-Band Radar Profiler Measurements in Italy

**DOI:** 10.3390/s23125524

**Published:** 2023-06-12

**Authors:** Mario Montopoli, Alessandro Bracci, Elisa Adirosi, Marco Iarlori, Saverio Di Fabio, Raffaele Lidori, Andrea Balotti, Luca Baldini, Vincenzo Rizi

**Affiliations:** 1 National Research Council of Italy, Institute of Atmospheric Sciences and Climate (CNR-ISAC), 00133 Rome, Italy; m.montopoli@isac.cnr.it (M.M.); a.bracci@isac.cnr.it (A.B.); elisa.adirosi@artov.isac.cnr.it (E.A.); 2 Center of Excellence Telesensing of Environment and Model Prediction of Severe Events (CETEMPS), 67100 L’Aquila, Italy; marco.iarlori@aquila.infn.it (M.I.); saverio.difabio@gmail.com (S.D.F.); raffaele.lidori@guest.univaq.it (R.L.); vincenzo.rizi@univaq.it (V.R.); 3 Department of Physical and Chemical Sciences (DSFC), University of L’Aquila, 67100 L’Aquila, Italy

**Keywords:** cloud radar, cloud and precipitation physics, remote sensing, dual-frequency radar techniques

## Abstract

Clouds cover substantial parts of the Earth’s surface and they are one of the most essential components of the global climate system impacting the Earth’s radiation balance as well as the water cycle redistributing water around the globe as precipitation. Therefore, continuous observation of clouds is of primary interest in climate and hydrological studies. This work documents the first efforts in Italy in remote sensing clouds and precipitation using a combination of K- and W-band (24 and 94 GHz, respectively) radar profilers. Such a dual-frequency radar configuration has not been widely used yet, but it could catch on in the near future given its lower initial cost and ease of deployment for commercially available systems at 24 GHz, with respect to more established configurations. A field campaign running at the Casale Calore observatory at the University of L’Aquila, Italy, nestled in the Apennine mountain range is described. The campaign features are preceded by a review of the literature and the underpinning theoretical background that might help newcomers, especially in the Italian community, to approach cloud and precipitation remote sensing. This activity takes place in interesting time for radar sensing clouds and precipitation, stimulated both by the launch of the ESA/JAXA EarthCARE satellite missions scheduled in 2024, which will have on-board, among other instruments, a W-band Doppler cloud radar and the proposal of new missions using cloud radars currently undergoing their feasibility studies (e.g., WIVERN and AOS in Europe and Canada, and U.S., respectively).

## 1. Introduction

Clouds cover about 70% of the Earth’s surface and are one of the most essential components of the global climate system. In fact, clouds strongly affect the Earth’s radiation balance regulating the planet’s average temperature by trapping heat near the surface and reflecting solar radiation back into space [1]. Such a cloud-competing radiative effect strongly depends on both the cloud macrophysical (e.g., coverage, frequency of occurrence, cloud vertical distribution, altitude, and thickness) and microphysical (e.g., phase, water and ice content, and hydrometeor size distribution) properties [2,3]. Nowadays, such properties are not adequately observed, with the twofold consequence of climate and weather forecast models suffering from a lack of sophisticated descriptions of cloud processes, while modellers do not have enough cloud observations to verify the models’ predictions [4,5]. Furthermore, because of their role in determining surface precipitation, clouds play a key role in the water cycle, redistributing water around the globe, thus replenishing the earthbound parts of the water cycle.

For all of the above, the importance of improving cloud and precipitation observation techniques is evident, in order to better quantify their parameters and characterize their behaviour in time and space. To this end, several cloud observation programs were launched.

In the U.S., the Department of Energy (DOE) established the Atmospheric Radiation Measurement (ARM) facility in 1992, considered the first organized effort to set up facilities able to provide continuously operating observatories for atmospheric studies using surface-based instruments [6,7,8]. ARM-supported measurement campaigns conducted in different climate regions have focused on the accurate high-resolution description of atmospheric columns with the ultimate goal to resolve uncertainties in climate and Earth system models. ARM observations are collected from in situ, remote sensing devices on fixed, mobile or airborne facilities, and the data are freely available. Among the instruments, cloud radars profilers are considered a key tool to support studies of the interactions between aerosols, clouds and precipitation processes.

At the same time, with the support of national weather services or research institutes, a number of atmospheric profiling observatories have been set up to establish formal collaborations with the DOE ARM program [9]. In particular, cloud radar profiling observations were only available in specific sites in France, Germany, UK, and Ireland. In 1998, the European Space Agency (ESA) funded the transversal field campaign CLARE’98 (Cloud Lidar and Radar Experiment) which involved lidars and radars over Europe in addition to the Chilbolton (UK) ground-based 94 GHz cloud radar [10]. The campaign stimulated a growing interest in Europe in research aimed at using observations of cloud processes to investigate their connection to aerosols.

This process led to the proposal of the Earth Clouds, Aerosol and Radiation Explorer (EarthCARE) satellite mission [11], which, at the time of writing, is scheduled to be launched in mid 2024. From 2001 to 2005, the CLOUDNET project [12], funded by the Fifth Framework Program of the European Union, provided a systematic tool over Europe to benchmark forecast models in terms of cloud process representations based on measurements collected by remote sensing systems. Similarly to initiatives in the U.S., lidar ceilometers, Doppler cloud radar, a microwave radiometer, and a disdrometer constituted the core instrumentation of such sites. These observatories have operated continuously for many years, collecting significant robust statistics related to a broad range of atmospheric events. Today, in Europe, the main efforts for collecting homogeneous observations of aerosols, clouds, and trace gases, is the Aerosols, Clouds, and Trace Gas Research Infrastructure (ACTRIS), a European research initiative supporting atmospheric research that provides tools to address current and future socioeconomic challenges, such as those related to air quality and climate change and protection from environmental hazards. It consists of eight central facilities, making an extensive network of observatories and high-level national facilities for atmospheric research spread across 21 European countries. In Italy, ACTRIS-IT (http://www.actris.it/index.php/en/) (accessed on 8 June 2023) [13] was lunched in 2015 and it now includes several research infrastructures involving research centres and universities across the country. One of them is located at the Casale Calore site, L’Aquila, Italy, belonging to the University of L’Aquila and managed by the Center of Excellence Telesensing of Environment and Model Prediction of Severe Events (CETEMPS). In August 2022, the observatory was updated with the installation of two new instruments, namely, a wind lidar and a W-band cloud radar. In this context, this article aims to introduce a joint field campaign conducted at Casale Calore, named the Combined Observations of Radar Experiments in L’AQuila (CORE-LAQ), which is the first effort, within the ACTRIS-IT community, to put multi-frequency radar profilers to use. CORE-LAQ was possible thanks to a collaboration between the National Research Council of Italy, the Institute of Atmospheric Sciences and Climate (CNR-ISAC) and CETEMPS. For this campaign, a micro-rain radar (hereafter termed a K-band radar or simply MRR) and a W-band cloud radar, operating at 24 GHz and 94 GHz, respectively, were placed close together. The main objective of the CORE-LAQ is to explore, for a specific climate region, the potential of multi-sensor measurements in deriving microphysical properties of precipitating clouds in rain and snow precipitation regimes. Particular emphasis is put on developing and validating methods for retrieving profiles clouds and precipitation along the atmospheric column above the Casale Calore site. The high-resolution vertical profile, particle classification, particle size distribution and vertical air motions are the main products expected form the campaign. Target applications focused on the Central Appennine region include the assimilation of numerical weather models [14] and the improvement of estimates obtained from operational scanning weather radar, highly sensitive to inhomogeneities along the vertical profile [15], especially in mountainous environments as known from past high-impact rain episodes [16]. This is worth highlighting as this type of measurement can be of particular interest for future satellite cal/val activities in light of incoming and future satellite missions involving spaceborne W-band radars, such as the mentioned EarthCARE [11], WIVERN (https://www.wivern.polito.it) (accessed on 8 June 2023) and AOS (https://aos.gsfc.nasa.gov/measurements.htm) (accessed on 8 June 2023), respectively.

This paper focuses on preliminary dual-frequency radar measurements collected during CORE-LAQ and the first results obtained concerning raindrop size spectra. The combination of K- and W-band measurements is still extremely rare and it allows, when processed in terms of Doppler quantities, to retrieve profiles that are not influenced by vertical air motion, calibration bias and signal attenuation. The results found indicate that the under-explored K- and W-band combination could be useful for microphysical retrieval of precipitation although some issues related to the differential sensitivity of the two systems could partially impair the applicability of the dual-frequency method. The dual Doppler difference retrieval of the raindrop’s mean size shows an RMSE of 0.3 mm within an interval of [0.5,1.5] mm.

The manuscript is organized into five sections. Section 2 gives a comprehensive overview of the theoretical background and a literature review of the techniques used to maximize the utility of cloud profiling radars. Section 3 describes the Casale Calore site, providing details on the instruments and tools available during the CORE-LAQ campaign. Section 4 shows the first example of combined K- and W-band radar measurements and their application to rain microphysics retrieval. Section 5 draws the conclusions and future perspectives.

## 2. Literature Review and Theoretical Background of Ground-Based Cloud Radar Potentials

This section aims to give a reference point concerning the most widespread estimation techniques that use ground-based cloud radar W-band profilers. Such techniques are herein classified as single-, double- and triple-frequency radar methods. The complementarity of cloud radars with other profilers, such as lidars, as well as passive microwave radiometers is shortly reviewed. In terms of applications, the reader can refer to Table 1 for a clear and concise view of the exploitation of W-band radar atmospheric profiling from published reports.

### 2.1. Single-Frequency Methods

One of the unique features of W-band radar profilers for spectral rain measurements, along with increased sensitivity to smaller particles than lower-frequency systems (i.e., an additional 17 dB of sensitivity with respect to the K-band due to frequency scaling in the Rayleigh scattering regime with the same transmit power and bandwidth), is the non-Rayleigh resonance signatures, i.e., an anchor point that coincides with a first characteristic spectral minima, found in the vertically observed Doppler spectra in still air and at sea level, at 5.9 ms${}^{-1}$ produced by the drop diameters D∼1.65 mm according to the well-established drop terminal velocity experimental laws [65]. A Doppler spectrum is the distribution of the return power over a range of Doppler velocities determined by the terminal velocity of drops and vertical air motion that shifts the Doppler spectrum to higher or lower velocities. A shift in the measured anchor point at the W-band with respect to the expected value can be easily detected and attributed to the the mean vertical air motion [41,42]. This method is simple to apply and, as long as the received radar signal is above the instrumental noise, is unaffected by radar signal path-attenuation effects that can severely affect the W-band. However, for the air velocity spectral retrieval technique to be applied successfully, particle sizes larger than 1.65 mm must be present in the radar beam, and spectrum broadening caused by air turbulence and/or multimodal drop size distribution (DSD) cannot dominate the received signal. The estimation of the vertical air velocity is not only important by itself, but it is fundamental to correctly retrieve the DSD with Doppler spectral methods. Whereas such techniques in rain are based on the assumption that drops fall at their terminal velocities [17,18]. For DSD retrievals, the following equation is used:

(1) $$S\left(v,r,\lambda \right)=\sigma_{b}\left(D,\lambda \right)N\left(D,r\right)\frac{dD}{dv}$$

Equation (1) has long been established [66] and expresses the link between the Doppler spectra (S(v,r,λ)) at a given wavelength (λ) and distance from the radar (*r*), and the drop size distribution (N(D,r)) through the radar backscattering cross-section ($\sigma_{b}\left(D,\lambda \right)$) of a rain drop of equivolume diameter (*D*) and terminal fall velocity (*v*). To invert Equation (1) to find N(D,r) the measured Doppler spectra (that can differ from the intrinsic S(v,r,λ)) have to be processed in order to ensure correct absolute system calibration, identify the rain region of a precipitating cloud, remove the effects of vertical air velocity that cause a shift in *v* and the consequent spectra, dealiase the Doppler velocity in case of Nyquist velocity folding, fix a unique and monotonic drop size–fall velocity relationship (v(r,D)) in stagnant air to be able to calculate the term dD/dv in (1), and, finally, identify any spectral broadening effects from turbulence that could limit DSD retrievals. The calibration of ground based W-band radar can be assessed using natural targets (e.g., exploiting the reflectivity factor plateau in light rain rate regimes [58]) compared with long-term satellite data statistics [59,60] or reference ground disdrometers [61]). The backscattering cross-sections of rain drops are typically calculated using readily available electromagnetic models (e.g., the T-matrix Python implementation [67]), whereas vertical air motion can be effectively identified using the spectral characteristic minima at the W-band, as previously described. Concerning the drop terminal velocity, the use of experimental laws [65], compensating for air density variations [68], is a common choice. A major limitation of DSD retrieval by the inversion of (1) is due to path attenuation (including gas, rain, and wet radome effects) that can be particularly severe in the W-band (order of 10 dB one-way for rain rates of 15 mm h${}^{-1}$ [69]). This causes fading of the spectral components S(v,r,λ), consequently introducing a multiplicative bias in N(D,r). As a result, a DSD retrieved from the measured (attenuated) spectra can only provide information about the shape of the DSD. An alternative single-frequency approach with respect to the inversion of Equation (1) is to minimize the quadratic difference between the measured Doppler spectrum and a reconstructed one, accounting for a particle’s backscattering properties, vertical air motion and turbulence effects. However, such an approach requires the a priori definition of a DSD model and can provide unrealistic results and artefacts [19,20]. Once the DSD has been defined, the relevant moments (i.e., liquid water content and rain rate) can be easily calculated.

### 2.2. Dual-Frequency Radar Methods

Dual-frequency radar methods are based on beam-matched observations of two radar systems working at different wavelengths. Three types of methods are described: reflectivity-, spectral-, and Doppler-based methods.

#### 2.2.1. Reflectivity-Based Methods

The basic idea that underlies the class of methods based on the dual-frequency ratio (DFR), also referred to as the dual-wavelength ratio (DWR), is that the pronounced non-Rayleigh backscattering at higher frequencies can provide, under negligible or compensated path attenuation effects, information on the characteristic size distribution (e.g., $D_{m}$, defined as the ratio of the fourth and third moment of the DSD). In particular, to define the DWR, we need to consider the equivalent reflectivity factor ($Z_{e}\left(r,\lambda \right)$), which is the quantity extracted from radar power measurements, typically expressed in $dBZ=10log_{10}\left({\mathrm{mm}}^{6}m^{-3}\right)$:

(2) $$Z_{e}\left(r,\lambda \right)=10log_{10}\left\{\frac{\lambda^{4}}{|K_{w}{\left(\lambda ,T\right)|}^{2}\pi^{5}}N\left(r\right)\int_{D_{min}}^{D_{max}}\sigma_{b}\left(D,\lambda \right)N_{shape}\left(D,r\right)dD\right\}$$

where, for convenience, the DSD term, N(D,r), is factorized into $N\left(r\right)N_{shape}\left(D,r\right)$. Different factorizations have been proposed for N(r) that denote the intercept parameter of the DSD [70] and $N_{shape}\left(D,r\right)$. If N(r) is the drop concentration number, $N\left(r\right)N_{shape}\left(D,r\right)$ can be interpreted as the probability distribution of particle sizes [71]. In addition, $|K_{w}{\left(\lambda ,T\right)|}^{2}$ is the dielectric factor of water at the radar wavelength and at a reference temperature (*T*). At *T* = 0 ${}^{\circ }$C, $|K_{w}{|}^{2}$ = 0.93, 0.90, 0.86 and 0.71 for the X-, K-, Ka-, and W-band, respectively. However, the measured reflectivity factor ($Z_{m}\left(r,\lambda \right)$) differs from ($Z_{e}\left(r,\lambda \right)$) by two-way path attenuation due to wet radome, gas and liquid hydrometeors. Focusing on attenuation due to liquid hydrometeors

(3) $$Z_{m}\left(r,\lambda \right)=Z_{e}\left(r,\lambda \right)-\underset{\begin{array}{c} \mathrm{two}\text{-}\mathrm{way}\\ \mathrm{path}\;\mathrm{attenuation} \end{array}}{\underbrace{2\int_{0}^{r}k(s,\lambda )ds.}}$$

where the term k(r,λ) represents the radar signal-specific attenuation in dB km${}^{-1}$, such that k(r,λ)dr is the one-way signal loss in dB in an atmospheric layer of thickness dr for a specific resolution at distance *r*. The DWR is simply defined as the ratio (i.e., difference in logarithm scale) of $Z_{m}\left(r,\lambda \right)$ at two different wavelengths, $\lambda_{1}$ and $\lambda_{2}$, with $\lambda_{1}>\lambda_{2}$ as

(4) $$\begin{array}{cc} DWR(r) & =\underset{\mathrm{differential}\;\mathrm{reflectivity}}{\underbrace{Z_{e}\left(r,\lambda_{1}\right)-Z_{e}\left(r,\lambda_{2}\right)}}+\\ \; & +\underset{\begin{array}{c} \;\mathrm{two}\text{-}\mathrm{way}\\ \mathrm{differential}\;\mathrm{attenuation} \end{array}}{\underbrace{2\int_{0}^{r}\left[k\left(s,\lambda_{2}\right)-k\left(s,\lambda_{1}\right)\right]ds}} \end{array}$$

We can see that the DWR has two components: the differential reflectivity and differential attenuation. Once the latter is quantified and subtracted from the DWR, the former has the noticeable property of being independent from the DSD intercept parameter N(r) in (2). Consequently, DWR is driven by $N{\left(r\right)}_{shape}$ such that, in the case of the gamma DSD model, it only depends on the shape factor μ and characteristic size parameter $D_{m}$. Once μ is fixed, the differential reflectivity component of the DWR is a direct function of $D_{m}$, thus allowing hydrometer sizing [72]. Such an approach was followed to develop the precipitation retrieval algorithms for the dual-frequency radar (Ku- and Ka-band) of the NASA/JAXA Global Precipitation Measurement (GPM) mission [73]. However, reliable particle sizing using DWR requires compensation for the differential attenuation. In the GPM radar algorithms, this is achieved by using the sufrace backscattering as a reference [73]. This technique is not straightforwardly applicable to ground-based radar and, in this case, reliable particle size retrieval is only possible if propagation is through ice or drizzle clouds where the attenuation effects are considered negligible and the non-Rayleigh regime holds (i.e., $Z_{e}\left(r,\lambda_{1}\right)\neq Z_{e}\left(r,\lambda_{2}\right)$). However, it is worth noting that when inter-calibration issues, the differential reflectivity term should be 0 dB in the Rayleigh scattering regime (i.e., the wavelengths $\lambda_{1}$ and $\lambda_{2}$ are much greater than the minimum particle size in the observed particle size distribution (PSD)); otherwise, the Mie scattering regime begins. This poses some constraints on the selection of wavelengths to be adopted that can guarantee a non-zero differential reflectivity term, thus extending the applicability of the DWR approach to lower $D_{m}$. In addition, the DWR-$D_{m}$ can suffer from multiple solutions in liquid regimes due to different resonant effects at $\lambda_{1}$ and $\lambda_{2}$, (e.g., see [74] for DWR in the Ku- and Ka-band frequencies). In spite of these difficulties, dual-wavelength approaches for ground-based radars have been successfully used for DSD retrieval. In this respect, it is worth mentioning the agreement of the simulated and measured spectra when tested using an iterative scheme [19] or optimal estimation framework, using the DWR to constrain the estimation procedure [20]. To date, the preferred pair of wavelength combinations used for ground-based radar systems is the Ka- (λ=8.6 mm) and W-band (λ=3.2 mm) for ice [21], particles sizing, DSD estimation [19,20,24], rain rate [49] and liquid water content [44,47,50,51] quantitative estimation. Other wavelength combinations that have been used include the X- (λ=3 cm) and W-band [27] and, more recently, K- and W-band [28,52], thus expanding the opportunities of further frequency combinations. It worth noting that, from space, the GPM mission is the first to adopt the dual-wavelength approach using the Ka- and Ku-band for DSD and precipitation parameter retrieval [74]. Future missions, such as AOS, will rely also on a dual-frequency approach using other combinations of bands.

#### 2.2.2. Spectral-Based Methods

A further extension of the DWR that enables handling situations with more pronounced attenuation, such as stratiform and moderate rain regimes, is the dual-spectral ratio (DSR) technique [75]. The DSR, for two wavelengths $\lambda_{1}$ and $\lambda_{2}$ is similarly defined to the DWR but in terms of the spectral ratio:

(5) $$DSR\left(v,r,\lambda_{12}\right)=\frac{S(v,r,\lambda_{1})}{S(v,r,\lambda_{2})}=\frac{\lambda_{1}^{4}}{\lambda_{2}^{4}}\frac{\sigma_{b}\left(D,\lambda_{1}\right)}{\sigma_{b}\left(D,\lambda_{2}\right)}$$

The great advantage of the DSR is that the Rayleigh and Mie scattering regimes can be disentangled by looking at some typical features. In particular, the DSR exhibits a Rayleigh plateau (i.e., constant DSR) for hydrometeor sizes sufficiently lower than $\lambda_{1}$, namely at lower terminal velocities. Such a plateau is particularly useful because when the Rayleigh regime holds, the DSR (as well as the DWR) is only dominated by the differential attenuation term (see Equation (4)). Thus, comparing the level of the DSR plateau at the cloud base, where differential attenuation is reasonably closer to zero, with higher altitudes provides an estimate of the differential attenuation term in (4) that can be removed, thus allowing proper retrieval of the differential reflectivity component of the DSR and $D_{m}$. This technique was successfully applied in a stratiform light rain event combining W- and Ka-band radars [75], but it is potentially suitable for drizzling stratocumulus and for higher rain rates, as long as the lower-wavelength received signal remains well above the noise level. In addition, the direct retrieval of the differential attenuation using the DSR has allowed the liquid water content to be estimated in stratocumulus clouds [44], mixed phase clouds [50] and mid-latitude rain precipitation clouds [49]. It is worth emphasising that the DSR is not applicable to airborne or satellite radar sensors, as platform movement introduces artefacts that irreparably broaden the Doppler spectrum.

#### 2.2.3. Doppler-Based Methods

Doppler spectra allow for the calculation of the mean Doppler velocity ($V_{D}$), obtained as the reflectivity-weighted terminal velocity of hydrometeors:

(6) $$V_{D}\left(r,\lambda \right)=\frac{\int_{D_{min}}^{D_{max}}\sigma_{b}\left(D,\lambda \right)N_{shape}\left(D,r\right)v\left(r,D\right)dD}{\int_{D_{min}}^{D_{max}}\sigma_{b}\left(D,\lambda \right)N_{shape}\left(D,r\right)dD}\pm w_{a}$$

where $w_{a}$ is the vertical air component that is generally unknown. Then, the difference between the mean vertical Doppler velocities (DDV) at two wavelengths is obtained as

(7) $$DDV\left(r,\lambda_{12}\right)=V_{D}\left(r,\lambda_{1}\right)-V_{D}\left(r,\lambda_{2}\right).$$

Note that Equation (6) can be easily written in terms of a Doppler spectrum using the relation in (1), cancelling the $w_{a}$ terms.

Similar to the DWR, DDV is related to $D_{m}$ in moderate rain regimes [23] and ice clouds [21]. Unambiguous retrievals of $D_{m}$ were tested using a Ka–W band radar combination with a particle diameter interval between 0.5 and 2.0 mm and between 1.5 and 4 mm for rain drops and ice particles, respectively. DDV is purely based on Doppler velocities giving it interesting properties: (i) immunity to differential attenuation and absolute radar mis-calibration, as both of these sources of uncertainties affect the received power rather than the phase from which $V_{D}$ is derived; (ii) less dependency on spectral broadening (compared to reflectivity-based methods), since spectral broadening typically has a symmetrical Gaussian distribution which does not affect the calculation of $V_{D}$; (iii) immunity to vertical air motions, which hamper the use of single-frequency vertically pointing Doppler radar-based retrievals of the DSD parameters [23]; (iv) For the DWR, DDV does not depend on the scaling parameter N(r) of the PSD (see Equation (6)) which is dependent on $D_{m}$. However, such techniques has their shortcomings that will be analysed later through experimental data. It is worth highlighting that the vertical Doppler velocity could also be used for particle sizing using a single-frequency. However, for single-frequency measurements the generally unknown contribution of the vertical air motion $w_{a}$ increases estimation uncertainties. Significant long-term averaging can nullify the net contribution of air motion, but at the expense of a significant reduction in the time resolution.

### 2.3. Triple-Frequency Radar Methods

From previous sections it is clear that dual-wavelength radar retrievals outperform single-frequency approaches, providing additional constraints to the PSD parameters. Triple-frequency approaches are particularly suited to study the microphysical properties of ice and snow particles. These particles usually show a large natural variability (e.g., PSD, density, and irregular shape) that results in uncertainty in the backscattering properties, and their characterization is difficult. Compared to the dual-wavelength approach, the addition of a third wavelength (typical configuration being the Ku-, Ka-, and W-band) allows for a characteristic curve almost independent from the PSD and velocity–dimension relationship with the potential of both distinguishing between different snowflake classes and improving the retrieval of the characteristic size or width of the PSD [76]. Reports using triple-frequency signatures in ice regimes can be found in [29,30,31,32,33,34]. Occasionally, triple-frequency radars were used in rain regimes to characterize the DSD [35]. However, it should be noted that although multi-frequency retrievals offer a more robust retrieval and ice process classification, it is less frequently used due to its cost and logistic maintenance barriers.

### 2.4. Complementarity with Other Instruments

As described in Section 2.2 and Section 2.3, the multi-frequency combination of cloud radars reduces ambiguity in the microphysical retrievals and, to some extent, in differentiating among particle types. Cloud radar reflectivity alone cannot be used to discriminate between liquid and ice as there is a significant overlap in the observed reflectivity values between liquid and frozen precipitation. Thus, the multi-frequency concept is often extended to lidar wavelengths (light wave emissions at wavelengths of around 1 μm) to generate a better and complete picture of the whole cloud characteristics, especially for the retrieval of particle types.

While cloud radars are sensitive to the vertical profile of hydrometeors (e.g., cloud droplets, rain drops, drizzle, frozen particles, etc.), they are less sensitive to small, non-precipitating liquid droplets. Lidars, which are in general much more sensitive to these small drops than radar, provide complementary observations of the cloud-base height and location of the drops (until the laser is extinguished). Such complementary is particularly significant when observing mixed-phase stratocumulus clouds in mid-latitude and polar regions because both supercooled liquid and frozen particles coexist in these clouds at temperatures well below freezing making their discrimination extremely challenging. Radar–lidar combinations allow for the effective discrimination of these particles into typical classes: aerosol, supercooled liquid drops, cloud droplets, drizzle, frozen particles and rain precipitation [12,53,77]. It is worth noting that [77] also provides a trimmed-down version of a fuzzy classifier algorithm that uses single-frequency 94 GHz airborne cloud radar fields to provide particle information. Other applications of radar–lidar synergy include turbulence and continuous profiles of the horizontal wind from near the surface to the cloud tops [78] as an accurate cloud boundary height estimate [79].

## 3. The Casale Calore Observatory in L’Aquila

**Figure 1:**
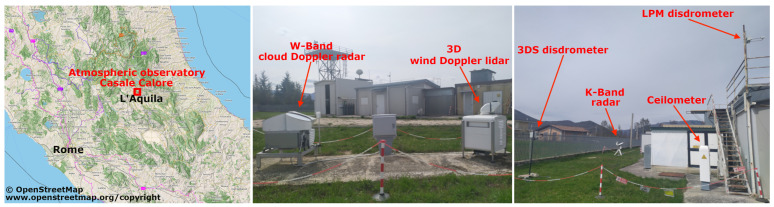
The Casale Calore site with the position of the instruments installed during the CORE-LAQ field campaign.

The Casale Calore observatory is located near L’Aquila, Italy (42.383081 N, 13.314806 E, 683 m ASL) surrounded by the Appenine range (see Figure 1). Casale Calore is the atmospheric observatory of the University of L’Aquila, hosted by the Department of Physical and Chemical Sciences and managed by the Center of Excellence Telesensing of Environment and Model Prediction of Severe Events. The observatory is situated in a valley with a temperate climate with warm summers and without a dry season (Köppen–Geiger-type Cfb) [80]. The annual mean temperature is 12.8 ${}^{\circ }$C (minimum temperature 5.9 ${}^{\circ }$C, maximum 19.6 ${}^{\circ }$C), and the annual precipitation is 620 mm distributed over 85 rainy days, with autumn being the rainiest season. The circulation in the valley is dominated by the mountain–valley breeze system, with anabatic up-valley (to the NW) during the daytime and katabatic down-valley (to the SE) during the night time [81]. The observatory hosts several instruments and routine activities to collect long records of ozone, pressure, temperature and water vapour. Recent funding opportunities have allowed to the site deploy new instruments, thus extending the observation capabilities to clear sky winds as well as cloud and precipitation profiles. Thanks to these updates, a field campaign, named CORE-LAQ, was set up and officially started on 13 December 2022 and expected to end in the summer 2023, thanks to a fruitful collaboration between CETEMPS and CNR-ISAC. CORE-LAQ focuses on cloud and precipitation observations made possible by simultaneous acquisition W- and K-band vertically pointing radars and disdrometers positioned close to the radars (Figure 1). The main goal of CORE-LAQ is to investigate the use of dual-frequency radar in the K- and W-band to retrieve cloud and precipitation microphysics. Such an investigation is quite unique and it is the first of its kind in Italy. After the conclusion of the campaign, the raw data acquired will be made freely available on a public repository.

### 3.1. K-Band Radar

The K-band (24 GHz) radar used in this study is mostly known as the Micro Rain Radar (MRR) PRO version, manufactured by METEK Meteorologische Messtechnik GmbH, Germany. It is a CNR-ISAC instrumentation and was moved to Casale Calore for use in the field campaign. It is a fixed vertically pointing antenna, single-polarized radar system that allows measurements of the Doppler spectra mainly caused by hydrometeor scattering. MRR is designed to primarily study the liquid precipitation such that its maximum unambiguous range is set to altitudes comparable to the expected annual freezing level variations (i.e., from 100 to 4500 m in our case). Acquisitions are performed every 10 s with a range resolution of 35 m using a single chirp. In addition to profiles of power spectra, the software provided by the manufacturer produces geophysical outputs such as rain rate, liquid water content and drop size distribution for the liquid phase. The MRR technical details can be found in Table 2.

### 3.2. W-Band Radar

The W-band (94 GHz) radar installed at Casale Calore is the RPG-FMCW-94 radar manufactured by the Radiometer Physics GmbH, Germany. It was purchased in mid 2022 by the University of L’Aquila during the ACTRIS-IT program. It is a vertically pointing frequency-modulation continuous wave systems with dual-polarization capabilities with high sensitivity to detect both cloud droplets and precipitating hydrometeors. The standard radar output quantities are the equivalent reflectivity factor, $Z_{e}$, mean vertical velocity ($V_{D}$), width (SW), skewness and kurtosis of the spectra linear depolarization ratio, (LDR), and co-cross-polar correlation coefficient ($\rho_{cx}$) as well as the spectra of *Z*, LDR, and $\rho_{cx}$. The W-band radar performs continuous acquisition every second covering altitudes from 100 to 10,000 m. The technical specifications as listed in Table 2. It uses three independent chirps to profile the atmosphere in three consecutive sectors and for each of them a specific Doppler resolution and dynamic range is achieved (see Table 3). In our case, the resolution range in the three vertical sectors is maintained at 30 m while the Doppler dynamics decrease with range. The system is also equipped with an 89 GHz radiometric channel and a weather station, whereby the liquid water path is internally estimated by the radar software.

### 3.3. Disdrometers

Two different disdrometers, belonging to CNR-ISAC, are installed at Casale Calore: the Laser Precipitation Monitor and the 3D-Stereo disdrometer, both manufactured by the Adolf Thies GmbH & Co. KG, Göttingen, Germany.

#### 3.3.1. Laser Precipitation Monitor

The Laser Precipitation Monitor (LPM) is designed to derive the PSD in different rain regimes (e.g., drizzle, rain) and provides hydrometer identification and the precipitation rate in hail, snow, or mixed precipitation. The precipitation category and intensity estimation rely on the obstruction of a laser beam (at a wavelength of 786 nm) from falling hydrometeors. The duration and amplitude of such is directly related to diameter and velocity of the falling particle, used both to derive the precipitation type through the relation between these two quantities and to derive the PSD. The raw output quantity of the instrument is a matrix reporting the number of falling particles for each particle diameter (rows) and velocity (columns) matrix entry. The number of rows and columns in the output matrix represents the diameter (20 classes, ranging from 0.125 to 9 mm) and velocity classes (22 classes, ranging from 0 to 12 ms${}^{-1}$), respectively. Nowadays, disdrometers in Italy are evolving in a network-like configuration allowing microphysical characterization of precipitation across the country [82].

#### 3.3.2. 3D-Stereo Disdrometer

The 3D-stereo (3DS) is a relatively new type of disdrometer, not yet analysed in the literature to best of our knowledge. Similar to LPM, it is able to detect PSD and identify precipitation type; however, the system uses a different measurement principle with a 3D detection ability. The instrument consists of a light source and a stereo camera. The measurement volume is defined by the viewing angles of the cameras as well as minimum and maximum distances from the cameras. Particles that passes through the measuring volume cause extinction of the light seen by the cameras. Particle sizes are deduced from the observed area by the cameras and their position within the measurement volume. Particle speeds are deduced from the movement of the particle during a predefined time. The raw data consist of a count matrix that provides, each minute, and the number of hydrometeors detected for each diameter and velocity class. The number of diameter and velocity classes is defined by the user. In this case we use 20 diameter and 22 velocity classes, as in the LPM, although the value of the class centres and widths differ from the classes in the LPM. The PSD and the type of precipitation, is derived from the count matrix (namely the velocity–diameter plane), similar to the LPM.

### 3.4. Other Instruments at the Casale Calore Site

At Casale Calore, several atmospheric quantities (ozone, pressure, temperature and water vapour) are routinely profiled. They are obtained by balloon-borne VAISALA radiosoundings that began in 1991 when a differential absorption lidar (DIAL) for aerosols and ozone measurements was active at the site [83]. The radiosoundings have continued since then, but a regular schedule (twice a month) for balloon-launching began in 2004 and is still active thanks to collaboration with the Ministero della Transizione Ecologica (Ministry of Ecological Transition) of the Italian Government. This allowed the creation of a continuously growing database, now populated with more than 300 thermodynamic atmospheric profiles which have been used for the retrieval of aerosol profiles from UV Raman lidar data in the EARLINET framework [84]. Since 2017, a CL-51 VAISALA ceilometer has been in operation 24/7 for vertical profile measurements of aerosols and clouds. In 2023, the ceilometer joined the EUMETNET/E-PROFILE network [85]. A Leosphere Windcube 100S wind lidar was also recently acquired. It has scanning capabilities and operates at the Casale Calore site to profile horizontal and vertical winds up to 3500 m in altitude. Both the celiometer and the wind lidar were bought within the ACTRIS-IT program [13].

## 4. First Dual-Frequency (K, W) Measurements during the CORE-LAQ Field Campaign

### 4.1. K- and W-Band Measurement Consistency

**Figure 2:**
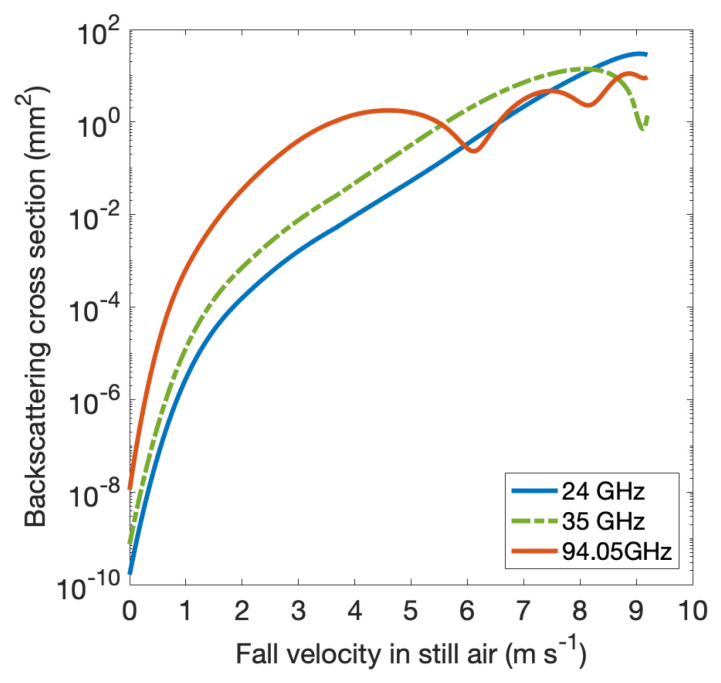
Simulated backscattering cross-sections at the K- (24 GHz), Ka- (35 GHz) and W-band (94.05 GHz) as a function of the fall velocity of water drops in still air at the sea level, 20 ${}^{\circ }$C temperature, 10${}^{\circ }$ drop canting angle using the Beard and Chuang drop size-shape model [86].

**Figure 3:**
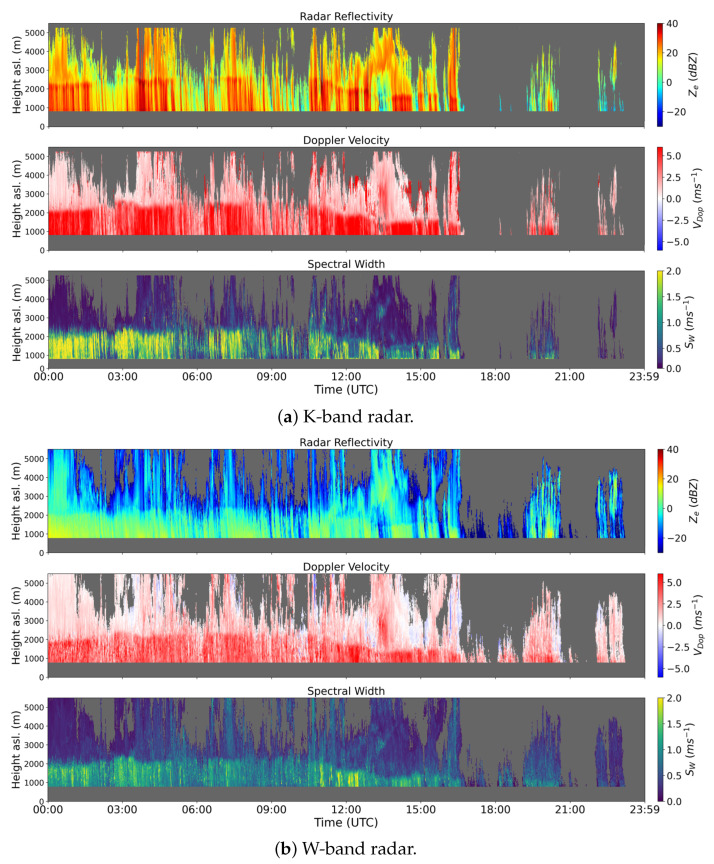
Example of K- (MMR-PRO) (**a**) and W-band (**b**) uncorrected measurements collected at Casale Calore observatory on 9 January 2023 between 00:00 UTC and 23:59 UTC. In each panel set, the radar reflectivity factor, Doppler velocity and spectral width are displayed from top to bottom, respectively. Note that in (**b**) the W-band radar range is limited to 5500 m, whereas the positive Doppler indicates a downward direction towards the radar.

CORE-LAQ has allowed to test, for the first time, the K and W-band combination in a rain regime. Theoretically, when used in combination with the W-band, the K-band has similar performances to Ka in terms of the backscattering properties. This is shown in Figure 2 in terms of the backscattering cross-section $\sigma_{b}\left(D\right)$ as a function of the drop terminal velocity. The two curves relative to the K- and Ka-bands are scaled to each other with no resonance effects for the K-band in the range of velocities (and diameters) depicted. This justifies the use of the K-band instead of the Ka-band, if a K-band radar is available along with a W-band radar. An example of actual measurements collected during the CORE-LAQ field campaign is shown in Figure 3. While the Doppler velocity is quite consistent between the two analysed frequency bands, the same cannot be said for the reflectivity factor, $Z_{e}$ (top subplots of (a) and (b)) or the spectral width ($S_{w}$), which depends on both the spread of the terminal velocities and the spectral broadening due to high cross winds and air turbulence effects. For $Z_{e}$, the difference is mainly due to the scaling effect of the term $\lambda^{4}/(\pi^{5}|K_{w}{|}^{2})$ in Equation (2), which explains about a 23 dBZ difference. In terms of spectral width, the lower values at the W-band are noted, likely caused by the Mie oscillations (Figure 2) which, in general, reduce the overall spectral broadening and/or by crosswind effects which have a more pronounced impact on the K-band system, possessing a beam width more than double that of the W-band (see Table 2).

### 4.2. K- and W-Band Dual-Doppler Velocity Retrieval

**Figure 4:**
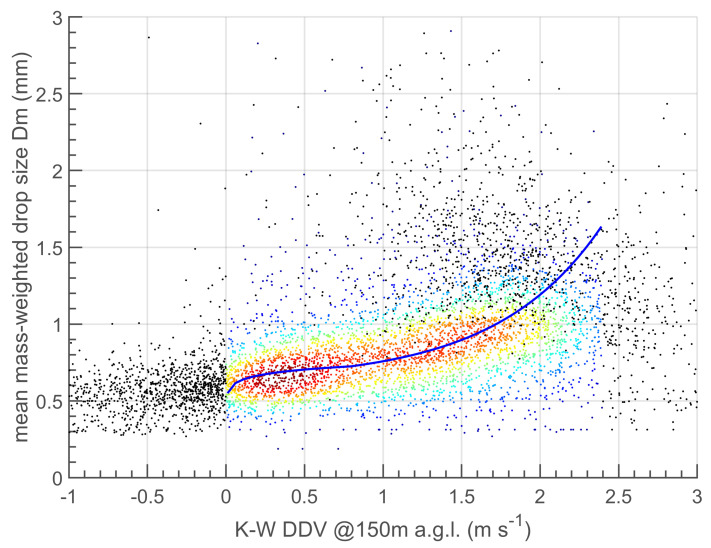
Density scatter plots for the K–W DDV vs. $D_{m}$ from the LPM disdrometer during the CORE-LAQ field campaign. Points are coloured based on data density ranging from dark red (high density) to dark blue (low density), whereas the black dots represents the filtered out data. The blue line is the regression curve (Equation (8) in the main text).

The preliminary analysis shown in Figure 2 and Figure 3 suggests an overall agreement of the vertically pointing K- and W-band combined acquisitions, thus suggesting the possibility of applying a series of methods already developed for the Ka–W band combination (see Table 1) and evaluating their performance when the Ka-band is replaced with the K-band. Among these methods, the one tested in this article is the DDV approach, as described by Equations (6) and (7). Here this method is implemented and evaluated for several case studies collected during the CORE-LAQ field campaign. The case studies consist of 6443 min of combined K–W acquisitions in a rain regime. One of the advantages of DDV is that the retrieval is neither influenced by the vertical component of the wind nor by the path attenuation and calibration issues. The mean Doppler velocity differences, obtained by a time series of K- and W-band Doppler spectra at the range gate of 150 m aloft, are compared with the $D_{m}$ obtained from the collocated measurements of the LPM disdrometer. The results of the comparison are shown in the density plot of Figure 4. From this figure an increasing trend of $D_{m}$ vs. DDV is noted although some doubtful samples are present (black dots). For small $D_{m}$, the Rayleigh regime should dominate and the power spectra tend to become similar for both wavelengths used, making DDV tend to zero. In our case, DDV does not tend to zero for $D_{m}$ smaller than 0.5 mm where we expect the Rayleigh scattering to hold, although the number of doubtful samples is not significant. This effects could be explained by the difference in the sensitivity of the K-band radar than that of the W-band radar. The K-band radar is much less sensitive than the W-band (see Table 2) and when approaching the sensitivity limit of the K-band system, the spectral components fade and the related Doppler velocity may unexpectedly vary causing a negative DDV, as shown Figure 4. Fortunately, the majority of the (DDV, $D_{m}$) pairs are distributed around zero DDV for small $D_{m}$, making the DDV vs. $D_{m}$ regression curve (blue curve in Figure 4) reasonably reliable. The quasi-vertical behaviour of the (DDV, $D_{m}$) points, for DDV larger than approximately 1.5 ms${}^{-1}$, is more problematic because it prevents any accurate retrieval of $D_{m}$ for large DDV. This is the main limitation of the DDV technique that was already noted in [23]. To avoid the inclusion of doubtful $D_{m}$ vs. DDV pairs in the retrieval of $D_{m}$, we filtered out data with DDV <0 ms${}^{-1}$ and >2.4 ms${}^{-1}$ as well as those with K-band $Z_{e}$ > 30 dBZ in order to remove heavy rain regimes. Consequently, the validity of the $D_{m}$ estimates is limited to [0.5, 1.5] mm. Finally, a double-fit-type (power-law and exponential) is used to obtain the regression curve resulting in the following relationships:

(8) $$\begin{array}{c} D_{m}=0.735DDV^{0.063}\left(\mathrm{for}\;\mathrm{DDV}\leq 1\;ms^{-1}\right)\\ D_{m}=0.633+0.092e^{1.5(DDV-0.8)}\left(\mathrm{for}\;1<\mathrm{DDV}<2.4\;ms^{-1}\right) \end{array}$$

The estimator in Equation (8) provides a root mean square error (RMSE) of 0.30 mm when tested on the CORE-LAQ dataset. Single-frequency Doppler estimators have been tested as well using the following estimators obtained from the CORE-LAQ dataset:

(9) $$\begin{array}{c} D_{m}=a\left(\lambda \right)V_{D}{\left(\lambda \right)}^{2}-b\left(\lambda \right)V_{D}\left(\lambda \right)+c\left(\lambda \right) \end{array}$$

**Figure 5:**
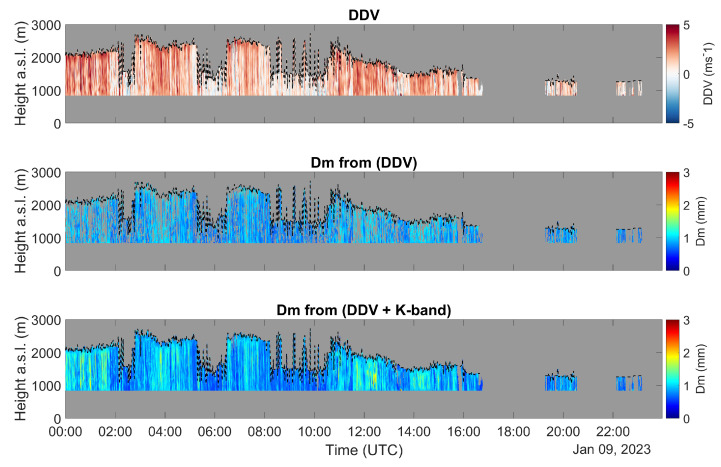
Time–height time series for the 9 January 2023 event of DDV (**top**); $D_{m}$ derived from DDV using Equation (8) (**middle**); and using a combination of DDV and $V_{D}$ from the K-band radar (Equations (8) and (9)) (**bottom**). The data shown refer to a rain regime only (i.e., areas below the melting layer altitude).

In (9) the wavelength-dependent regression parameters (a,b,c), are equal to (0.0203, 0.02729, 0.5228) and (0.08271, 0.2927, 0.7887) for λ=12.5 mm (K-band) and λ=3.2 mm (W-band), respectively. The use of (9) provides RMSE = 0.30 mm and 0.24 mm, at the K- and W-band, respectively. However, single-frequency relationships can be affected by vertical wind effects which are likely to not dominant in our dataset, mostly comprising winter stratiform events, thus leading to comparable or even better performances of the single-frequency W- and K-band estimators with respect to DDV. It is worth noting that the W-band offers the opportunity for vertical wind self-retrieval, overcoming the limitations of the single-frequency estimator; however, this is constrained to the presence of drops larger than 1.65 mm. Figure 5 shows an example of the K–W DDV (top) and the related $D_{m}$ product (middle) for the case study of 9 January 2023 and for a rain regime only. Some gaps can be noted in the height–time series of the retrieved $D_{m}$ caused by the limitation of Equation (8) for $D_{m}$ for [0.5, 1.5] mm. To maximise the columnar coverage and provide a more homogeneous product, those gaps are filled with K-band retrieval using (9) (bottom).

### 4.3. Limitations and Comparison with Previous Literature Results

The results presented in the previous subsection follow the same approach originally proposed by [23], who performed an analysis similar to ours using Ka- and W-band radars and a Parsivel disdrometer. However, some differences are noteworthy. In [23], the DDV collapsed to zero for $D_{m}<0.5$ mm, suggesting a much better radar sensitivity of their radar systems than ours. Similar to our results that produce K–W band DDV-based estimates of $D_{m}$ within [0.5, 1.5] mm, ref. [23] highlighted that the DDV based on the Ka–W band difference is valid for moderate rain regimes with $D_{m}$ within [0.5, 2] mm after filtering out the data for $V_{D}\left(Ka\right)$ > 6.9 ms${}^{-1}$. Finally, it is important to mention that the benefits brought by the DDV technique in terms of its immunity to some undesired and harmful effects, should be balanced with the applicability limitations in terms of the dynamic interval of the retrieved $D_{m}$. However, for radar system calibration purposes, it is still worth having unbiased estimates of $D_{m}$ through DDV for some reference intervals of $D_{m}$.

## 5. Conclusions

In this article, combined profiling radar measurements for atmospheric precipitation studies was presented. This is the first time that a W-band radar profiler has been available for atmospheric studies in Italy, owing to the recent funding opportunities obtained at the University of L’Aquila as part of the ACTRIS-IT program. This opportunity has set up a field campaign conducted through a joint collaboration between CETEMPS of the University of L’Aquila and CNR-ISAC. From this collaboration a dual-frequency radar experiment based upon K- and W-band frequencies was possible and a preliminary demonstration analysis, both theoretically and experimentally, was carried out. Although the results are not definitive, relevant indications have emerged. Theoretical simulations highlighted that the K-band is not expected to introduce much difference if it replaces the frequently used Ka-band in dual-frequency experiments with W-band radars for liquid drops. Nevertheless, experimental evidences show that one limiting factor in exploiting the dual-frequency approaches is related to the difference in the radar receiver sensitivity. In the analysis, the K-band radar had a much lower sensitivity than the W-band radar, thus producing undesired, but recoverable, signatures in the DDV approach. The latter allowed unbiased retrieval of $D_{m}$ in a limited range of diameters in the range of [0.5 1.5] mm for the K–W band configuration. Future wok plans to maximize the field campaign (still running at the time of the writing this article), analysing in more detail the retrieval techniques developed for the Ka–W band configuration, both in rain and snow fall regimes. This also will enhance the awareness, in terms of performance, of microphysical retrieval, when small K-band radar systems are manageable and widespread.

## Figures and Tables

**Table 1:** List of works which make use of ground-based W-band radars. The first letter in each entry of the description refers to the frequency band used.

Applications	Output Variables	Description
Cloud microphysics	Profiles of PSD in rain and ice Profiles of characteristic drop size ($D_{m}$) Size, number concentration and bulk density of falling snow Relevant physical processes (ice growth, supercooled droplets, riming, aggregation)	○ W. Retrieval of the vertical air motion, DSD and shape parameters using non-Rayleigh Doppler spectra inversion [17,18]. ○ Ka–W. DSD profiles from Doppler spectra for mean volume rain drop diameter larger than 1 mm [19,20]. ○ Ka–W. Differential Doppler velocity (DDV) and dual wavelength ratio (DWR) for sizing particles in thick ice clouds [21]. ○ Ka–W. Study of the influence of ice hydrometeor shape on DWR [22]. ○ Ka–W-band differences in mean DDV to estimate $D_{m}$ in rain regimes [23]. ○ Ka–W. Combined radar retrieval of DSD to evaluate the representation of rain microphysics in the WRF model [24]. ○ Ka–W. Neural network estimation of the riming fraction and supercooled liquid drops [25,26]. ○ X–W. Retrieval of snow microphysics [27]. ○ K–W. Observational fingerprints to identify ice crystal growth by aggregation of individual ice crystals and conditions for large snowflake production [28]. ○ X–Ka–W. Doppler spectra for a snowfall event to distinguish transition from rimed to unrimed snow aggregates [29]. ○ Ku–Ka–W. Optimal estimation of precipitating rain, ice and snow [30,31,32]. ○ Ku–Ka–W. Bayesian retrievals of the size, number concentration and density of falling snow [33]. ○ S–Ka–W. Ice PSD from spectral profiles [34]. ○ X–Ka–W. Retrieval for characteristic raindrop size and width of the rain drop size distribution (DSD) [35].
Cloud macrophysical properties	Cloud occurrence fraction, vertical distribution, persistence in time, diurnal cycle, and boundary statistics (e.g., layer base, layer peak, cloud base, cloud top).	○ Standard cloud type and macrophysical properties were identified by combining lidar, millimetre-wave radar, and radiometer measurements [36,37,38,39,40].
Cloud dynamical properties	Vertical air motion	○ W-band use of non-Rayleigh backscattering minima in Doppler spectra (expected at 5.95 ms${}^{-1}$ for falling raindrops at the surface level) as an anchor point to track the vertical air motion [41,42]. ○ Optimal estimation for cloud microphysics [20].
Quantitative precipitation estimation	Liquid water content, ice water content, liquid water path, rain rate, snow fall rate	○ Scattering and absorption characterization by cloud and precipitation from 35 to 240 GHz [43]. ○ Ka–W. Retrieval of liquid water content (LWC) in stratocumulus clouds (particles size lower than 0.6 mm) using the differential attenuation (i.e., dual-wavelength ratio in Rayleigh plateau regimes only) measured by vertically pointing radars [44]. ○ Retrieval of ice water content from multi-frequency radar [45,46]. ○ Ka–W. Total variation regularization technique to estimate cloud LWC [47]. ○ Optimal estimation of liquid and/or ice water content [20,48]. ○ Ka-W. Rain rate and optimal DSD parameter estimation from combined spectral profiles [49]. ○ Ka–W.Estimates the differential path-integrated attenuation (ΔPIA) and characterize cloud liquid water path (LWP) accordingly [50]. ○ Ka–W. Smoothed LWC dual-wavelength ratio in shallow clouds [51]. ○ K–W. Snowfall rate retrieval [52].
Cloud classification	Category map with indication of insects, aerosol, ice, melting ice, cloud droplets, supercooled droplets, drizzle, rain.	○ Use of radar, lidar and radiometer instrument synergy for target categorization. Radar is sensitive to large particles (e.g., rain, drizzle drops, ice particles, and insects) while lidar is sensitive to higher concentrations of smaller particles (clouds, supercooled droplets and aerosol). Thus, lidars enable the identification of supercooled liquid layers in mixed phase clouds. Sharp changes in the Doppler velocity close to the freezing level is a proxy for melting ice. The microwave radiometer (alternative to radiosondes) is used to continuously profile the cloud temperature [12,53].
Precipitation and fog forecast	Fog identification, fog thickness, fog LWP	○ Ground-based remote sensing for fog [54]. ○ W. Radar and radiometer observations for 1D VAR assimilation scheme of LWC [55].
Calibration	Short- (event-based) and long-term (multiyear-based) bias of the reflectivity factor.	○ Used reflectivity factor differences (in dB) in Rayleigh regimes (e.g., small ice at cloud top) from two radar systems operating at two separate wavelengths to identify miscalibration bias of one system with respect to the other [50,56,57]. ○ W. Exploitation of the 19 dBZ reflectivity factor plateau at 250 m aloft for rain rates between 3 and 10 mm h${}^{-1}$, as a reference calibration signature. A departure from the reference dBZ level gives the bias [58]. ○ W. Spaceborne cloud profiling radar (CloudSat) characterized the calibration of the ground-based cloud radars [59,60]. ○ W. (a) Radar calibration, propagating the simulated reflectivity factor in light rain regimes, derived from a ground-based disdrometer, at a radar range gate aloft, taking wind shear and drop evaporation into account. Then, the simulated and measured reflectivity factor were compared. (b) Extension of the self-consistency approach to W-band radars using *Z*, $Z_{dr}$ and $\Phi_{dp}$ at slanted incidence angles [61].
Cloud–aerosol interaction	Aerosol precursors and modification of cloud radiative properties.	○ Observational evidence (using a W-band radar) of aerosol emissions influencing the warm cloud microphysics and cloud–aerosol interactions [62,63].
Propagation effects	Channel availability statistics	○ Large available bandwidth at the W-band and onward for satellite and deep-space communications [64].

**Table 2:** K- and W-band cloud radar technical specifications and settings.

Specifications	Units	K-Band	W-Band
Frequency	(GHz)	24.23	94
Chirp repetition frequency	(kHz)	Data	Data
Doppler velocity resolution	(ms${}^{-1}$)	0.05–6	(0.041, 0.080)
3 dB Beam width	(${}^{\circ }$)	1.5	0.56
Nyquist velocity	(ms${}^{-1}$)	12.3	(±5.1, ±10.5)
Range resolution	(m)	35	$\tilde{30}$
Temporal sampling	(s)	10	1
(Min., Max.) range	(km)	(0.1, 4.5)	(0.1, 10)
Minimum detectable reflectivity	(dBZ)	−8	−47 (at 4 km agl.)
			−36 (at 10 km agl.)

**Table 3:** Configuration parameters used by the W-band radar for each chirp sequence.

Attributes	Units	Chirp Sequence		
		1	2	3
Range interval	(km)	(0.1, 1.233)	(1.233, 5.037)	(5.037, 10)
Range resolution	(m)	29.8	30.4	31.1
Nyquist velocity	(±ms${}^{-1}$)	10.5	7.9	5.1
Doppler velocity resolution	(ms${}^{-1}$)	0.041	0.062	0.080
Temporal sampling	(s)	0.156	0.388	0.462

## Data Availability

Data will be made available at the end of the field campaign.
